# A Study on the Impact of Temperature on the Anchoring Durability of Carbon-Fiber-Reinforced Polymer Cables

**DOI:** 10.3390/ma18020410

**Published:** 2025-01-16

**Authors:** Minzhe Wang, Bo Chen, Haozhe Jiang, Ping Zhuge

**Affiliations:** 1 School of Civil & Environmental Engineering and Geography Science, Ningbo University, Ningbo 315211, China; wmz13511399719@163.com (M.W.); jianghaozhenbu@163.com (H.J.); 2 Zhejiang Design Institute of Water Conservancy & Hydro-electric Power Co., Ltd., Hangzhou 310002, China; chenbo8095@163.com

**Keywords:** CFRP anchorage, durability, temperature effect, interface residual bond strength, Arrhenius theory

## Abstract

To improve the application of carbon-fiber-reinforced polymers (CFRPs) in civil engineering, the long-term durability of CFRP anchorage systems has become a critical issue. Temperature fluctuations can significantly impact the bond performance between CFRPs and the load transfer medium (LTM), making it essential to understand the effects of temperature on the durability of CFRP anchorages. Therefore, this study investigates the influence of temperature on the durability of CFRP anchorages through aging tests on 30 epoxy-filled CFRP-bonded anchorage specimens, followed by pull-out tests. The long-term degradation of CFRP cable anchorage performances in representative regions of the globe was predicted using Arrhenius theory. The experimental results show that after long-term temperature exposure, the maximum bond strength of the CFRP-LTM interface in the anchoring zone degrades after 30 days but continues to increase after 150 days. In contrast, the residual bond strength of the CFRP-LTM interface in the anchorage zone continuously decreases over time, with the degradation rates gradually decreasing over time. Higher temperatures lead to more severe degradation of anchoring performance. Based on the experimental results, it is predicted that the anchoring performance of a CFRP cable anchorage system will reach degradation rates of 63.72%, 83.36%, and 94.73% after 50 years in regions with average annual temperatures of 0 °C, 10 °C, and 20 °C, respectively. Therefore, the temperature has a significant long-term impact on the anchoring performance of CFRP cable bonding systems, necessitating a more conservative design in higher-temperature areas.

## 1. Introduction

Carbon-fiber-reinforced polymers (CFRPs) are characterized by their light weight, high strength, and excellent corrosion resistance, significantly addressing issues associated with traditional steel cables, such as their susceptibility to corrosion and poor fatigue performance [1,2,3]. Compared to their traditional steel rebars, CFRP cables offer the following advantages: (1) CFRPs exhibit strong corrosion resistance, with negligible performance degradation under acidic, alkaline, saline, and freeze–thaw conditions [4,5,6]. (2) The tensile strength of CFRP cables exceeds that of steel cables, meeting the load requirements for ultra-long cables. However, due to CFRP cables’ significant anisotropy (i.e., the transverse tensile strength is less than 10% of the longitudinal tensile strength), optimizing these advantages largely depends on the cable’s anchorage system [7,8]. Currently, the main types of CFRP anchorage devices include bonded anchorages [9,10], mechanical anchorages [11], and hybrid anchorages [12,13]. Hybrid anchorages combine the benefits of both mechanical and bonded anchorages, although their assembly process is relatively complex. Compared to mechanical anchorage systems, bonded anchorage systems are better suited for CFRP cables and offer wider application potential in cable structures.

Bonded anchorages typically consist of an anchorage cylinder and a load transfer medium, exhibiting better anchorage performance and fatigue resistance compared to mechanical anchorages. Some researchers have conducted pull-out tests on the bonded anchorage systems of CFRP cables at room temperature, assessing their anchorage performance and the bonding effect between the CFRP cable and the bonding medium [14,15,16]. The results indicate that the mechanical properties of the bonding medium significantly influence the interaction of and friction between CFRP cables and LTM interfaces, making it one of the key factors affecting the anchoring performance of bonded anchorage systems. Currently, the most commonly used bonding media in bonded anchorage systems are resin-based materials and cement-based materials. Compared to cement-based bonding media, resin-based bonding media have higher bonding strength but poorer high-temperature resistance. The interface between CFRP cables and the bonding medium is prone to fire, which also limits the further development of bonded anchorage systems in cable structures. In this context, Xu et al. [17] investigated the fire resistance of a high-temperature-resistant (HTR) CFRP tendon and showed that the tensile strength retention of the CFRP cables was 19% at 600 °C and that high initial stress (stress level of 0.57) exacerbated the damage to the CFRP tendon at high temperatures. Jiang et al. [18,19] investigated the performance of CFRP cables under high-temperature conditions. The results showed that the effect of a high temperature on the modulus of elasticity was more significant than that on the tensile strength. They also established a prediction model for the creep strain of CFRP cables at high temperatures. The fire resistance limits of CFRP cables with pre-tension ratios of 0.2~0.6 ranged from 3.26 to 15.40 min, and their fire resistance properties were poor. It is necessary to improve their fire resistance to expand their engineering applications.

In a study on the durability of CFRP anchoring systems, Hao et al. [20] studied the durability of CFRP cable anchoring systems in marine environments, identifying five stages of axial force and shear stress changes over time. They also recommended the optimal placement angles for CFRP cable anchors in offshore pit engineering. Ai et al. [21] found that self-anchored CFRP systems exhibit minimal creep and relaxation, maintaining excellent residual tensile properties. Xian et al. [22] developed a pre-stressed stretching device for CFRP plates, assessing the stress loss in anchorage systems under coupled temperature, humidity, and sustained load conditions and predicting the long-term tensile strength of CFRP plates. Pan et al. [23] evaluated the effects of corrosion damage and sustained loading on CFRP-reinforced RC beams, finding significant reductions in the load-bearing capacity under severe corrosion. Xie et al. [24,25] conducted extensive research on the durability of adhesive-type CFRP anchoring systems under various environmental factors. Their studies revealed that cyclic loading at frequencies above 12 Hz causes temperature rises within the anchorage system, leading to irreversible damage. Furthermore, they demonstrated that the water absorption increases with an early immersion time, weakening the bonding medium’s mechanical properties, including Young’s modulus, tensile strength, and interfacial fracture toughness. These deteriorations contribute to the degradation of the anchorage system’s tensile capacity, highlighting the critical impact of environmental factors on the long-term performance of adhesive-type CFRP anchorages. Zhu et al. [26] found that while water immersion and cyclic loading deform internal tendons, they do not affect the anchorage performance or cause corrosion. Similarly, Feng et al. [27] highlighted that the stress range significantly impacts the fatigue durability, with a linear axial strain under cyclic loading.

Despite these advancements, this literature review highlights a significant gap in our understanding of the mechanical properties of CFRP adhesive-type anchorages under long-term temperature effects, even though temperature is a critical factor influencing their durability. The environmental temperature adversely impacts the bond performance at the CFRP-LTM interface of adhesive anchorages, which is vital for the overall anchorage performance [28]. Given this gap, further investigation into the degradation of bond performance at the CFRP-LTM interface under prolonged temperature exposure is necessary.

To better evaluate the mechanical performance of CFRP cable-bonded anchorage systems using epoxy resin as the bonding medium under long-term temperature effects, this study conducted accelerated aging tests on 30 CFRP cable-bonded anchorage specimens. These specimens were subjected to elevated temperature conditions to simulate long-term exposure. Subsequently, pull-out tests were performed to measure the degradation rates of the maximum and residual bonding strengths at the CFRP cable–LTM interface under varying temperature conditions. By integrating performance assessment methods with the Arrhenius durability model, this study aimed to assess the long-term degradation of anchorage performance due to temperature exposure. Additionally, it provided predictions of the temperature-related durability of CFRP cable anchorages across representative climatic regions worldwide. The results presented here are expected to contribute to improving the reliability and durability of CFRP adhesive-type anchoring systems in diverse environmental conditions.

## 2. Bond Strength Assessment Model

The pull-out force *F* that is required for the specimen at the ultimate state of the anchorage interface is given by the following equation:

(1) $$F=\pi d_{c}l_{a}\tau_{a},$$

where $d_{c}$ represents the diameter of the CFRP reinforcement; $l_{a}$ refers to the effective anchorage length of the anchorage; and $\tau_{a}$ is the average bond strength at the interface, which is composed of the adhesion force $\tau_{e}$, the interlocking force $\tau_{b}$, and the friction force $\tau_{f}$.

**Figure 1:**
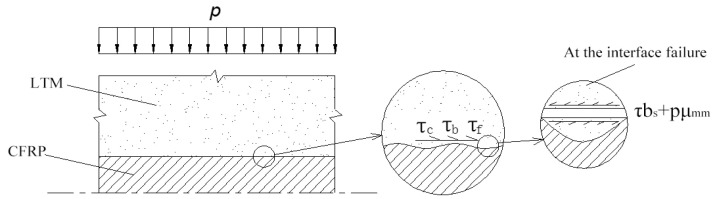
Schematic diagram of the interface force transfer mechanism.

If a compressive stress *p* is applied at the interface, the maximum bond strength at the interface just before failure can be expressed as follows:

(2) $$\tau_{max}=\tau_{e}+\tau_{b}+\tau_{f}=\tau_{e}+\tau_{bs}+p\left(\mu_{mm}+\mu_{cm}\right),$$

where $\tau_{bs}$ represents the average shear strength of the epoxy resin at the interface, $\mu_{mm}$ is the friction coefficient at the failure interface when the gel is sheared, and $\mu_{cm}$ is the friction coefficient at the moment of initial failure. The specific interface force transfer mechanism is shown in Figure 1.

From Equation (2), it can be seen that $\tau_{max}$ primarily depends on the surface geometry of the CFRP cable, the compressive stress at the interface, and the material properties of both the reinforcement and the epoxy resin.

The stress distribution at the CFRP-LTM interface of bonded anchorages is quite complex, making it difficult to reliably measure the load capacity of the conical CFRP cable anchorage system. During the pull-out process, in the initial stage of loading, there is almost no relative displacement between the CFRP reinforcement and the transfer medium, and the bond strength at their interface is primarily composed of adhesion and interlocking forces. As the tensile force increases, it reaches the ultimate state, at which point relative sliding occurs, leading to failure. The loading then enters the second stage, where residual bond strength still exists, typically greater than 30% of the peak interface stress. Thus, it is proposed that the residual bond strength $\tau_{res}$ can be used to assess whether the anchorage system can provide reliable anchoring capacity, with a linear relationship between the retention rate of the residual bond strength and the retention rate of anchorage capacity.

The value of $\tau_{res}$ is influenced by the surface geometry of the CFRP reinforcement, the mechanical properties of the transfer medium, and the radial compressive stress at the interface. Zhuge [29] proposed that the elastic elongation δ of the CFRP reinforcement in the anchorage zone under ultimate loading conditions can conservatively determine the residual bond strength $\tau_{res}$. The relationship between the residual bond strength and radial compressive stress can be expressed as follows:

(3) $$\tau_{res}=\left\{\begin{array}{cc} 0.088\sigma_{tm}+6.996 & \left(0\leq \sigma_{tm}<154\;\mathrm{MPa}\right)\\ 20.9 & \left(154\;\mathrm{MPa}\leq \sigma_{tm}\right), \end{array}\right.$$

where $\sigma_{tm}$ represents the radial compressive stress on the anchorage.

The residual bearing capacity $F_{res}$ can be expressed as follows:

(4) $$F_{res}=\int_{0}^{l}n\eta \pi d_{cu}\tau_{res}\left(x\right)dx,$$

where n represents the number of CFRP strands; $d_{cu}$ is the diameter of the CFRP filaments; $\tau_{res}\left(x\right)$ is the residual shear stress at position x; and η is the reduction coefficient, which accounts for the uneven tension between CFRP strands [30].

## 3. Experimental Program

### 3.1. Raw Materials

**Figure 2:**
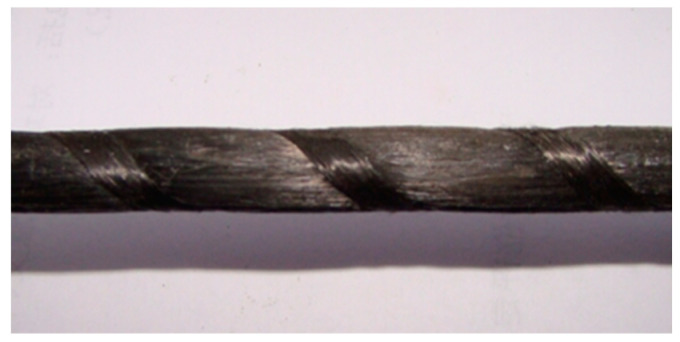
Textured CFRP cable.

The CFRP cables used in this study have a textured surface, as shown in Figure 2, which is also the most commonly used form of CFRPs in engineering applications. Their material properties and the properties of the Si-kadur 42 epoxy resin that was used as a bonding medium are listed in Table 1 and Table 2, respectively. The CFRP cables that were used in this study were provided by Aslan Company (Toledo, OH, USA), and the epoxy resin adhesive was supplied by China Zhonglan Chenguang Chemical Research Institute Fine Chemical Company(Chengdu, China).

### 3.2. Specimen Fabrication and Assembly

**Figure 3:**
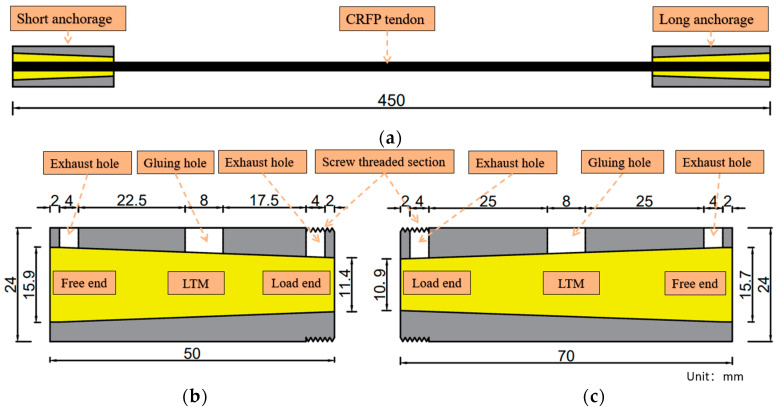
Specimen: (**a**) overall schematic diagram; (**b**) anchorage A; (**c**) anchorage B.

Based on the research by Guo et al. [28], the average bond stress measured at an anchoring length of 50 mm for textured CFRP anchorages indicates the maximum bond strength that is achievable at that interface. The CFRP system consists of a short anchorage (A), a long anchorage (B), and the CFRP cable itself. In this experiment, the A and B anchorages were made of 40CR material, and a conical anchorage tube was selected for the structure, which is the most common type in bonded anchorage systems. The 50 mm length of anchorage A was chosen to ensure that the short anchorage failed first. Anchorage B had a length of 70 mm, and considering the dimensions of the universal testing machine and other instruments that were used in the experiment, the CFRP cable extended to 450 mm. Detailed dimensions are provided in Figure 3. The upper part of the anchorage had one gluing hole and two exhaust holes for the injection of the bonding medium, and the external threads allowed the connecting nut to act as a force-transmitting medium when applying the preload force.

**Figure 4:**
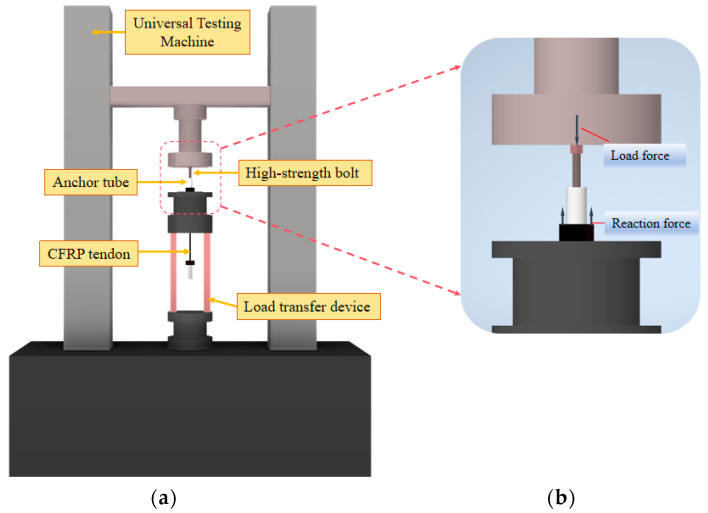
Test diagram of specimen preload application: (**a**) overall diagram; (**b**) schematic diagram of load transfer.

For textured CFRP anchorages, when the surface compressive stress reaches 100 MPa, the ultimate strength of the CFRP anchorage is achieved [31]. The anchorage zone in this experiment employed conical anchor pipes. Due to the small angle, the surface compressive stress can be approximated using a straight anchor tube calculation. A load of 22 kN was applied to the free end of anchorage A through high-strength bolts, resulting in an average radial compressive stress of 100 MPa. To induce failure at the short anchorage end, a load of 28 kN was applied to the free end of anchorage B. The preload was applied using displacement control at 4 mm/min, as shown in Figure 4.

### 3.3. Experimental Set-Up

**Figure 5:**
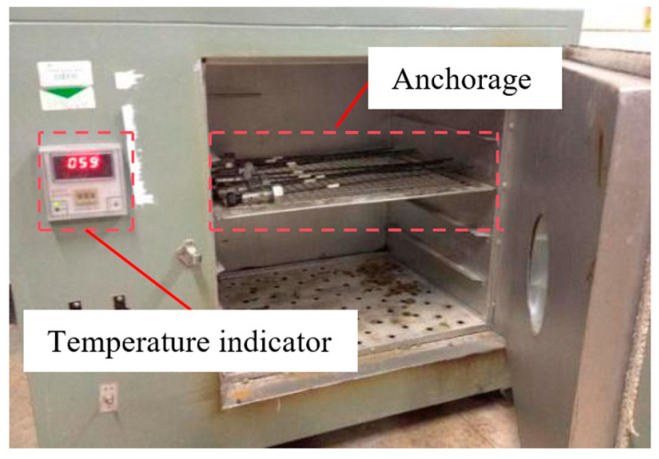
Temperature-accelerated aging test.

**Figure 6:**
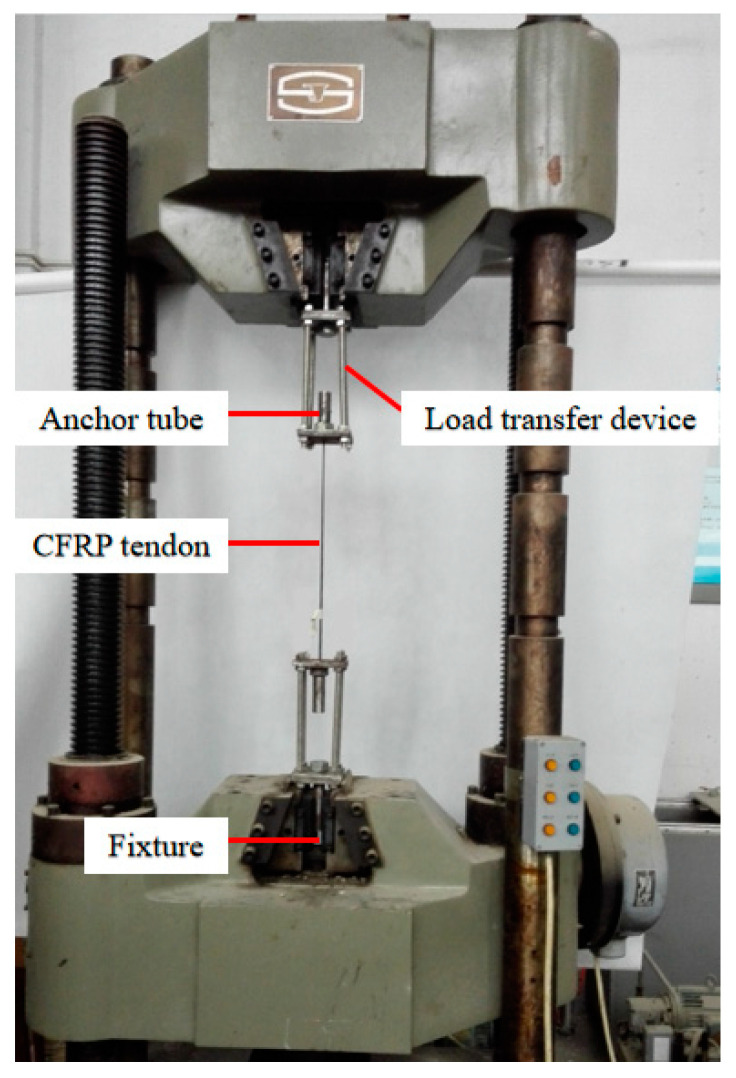
Pull-out test.

The experiment was conducted in two phases. First, the assembled specimen was placed in an oven for accelerated aging at a controlled temperature, as shown in Figure 5. After the accelerated aging test, a tensile test on the anchorage was performed using a universal testing machine (as shown in Figure 6) to measure the maximum and residual bonding strengths.

According to the experimental requirements, 30 CFRP conical steel tube anchorages filled with epoxy resin were fabricated for testing. The temperature aging conditions of 50 °C to 70 °C were selected based on relevant standards such as the European Standard (EN)-EN 14651:2005 [32] and ASTM D5229 [33]. Since this was a standalone temperature aging test, humidity control was not applied, and in this case, the humidity was determined based on the ambient air moisture in the testing environment. The accelerated aging test conditions and their corresponding identification numbers are provided in Table 3.

## 4. Test Results and Discussion

### 4.1. Control Group

The specimens in the control group were cured at room temperature and humidity and were subjected to a pull-out test after one month.

The ultimate load capacity $F_{max}$, maximum bond strength $\tau_{max}$, residual load capacity $F_{res}$, residual bond strength $\tau_{max}$, and failure modes of each specimen are presented in Table 4.

**Figure 7:**
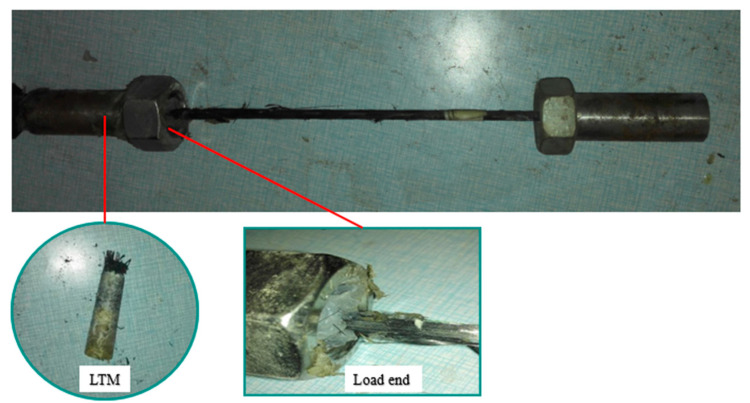
Slip failure diagram of the short anchorage end of the BS-t1-B specimen.

After the standard aging process, the ultimate load capacities of the three specimens were generally consistent, and the colloid maintained good adhesion integrity. As shown in Figure 7, the failure modes were identical for all the specimens exhibiting slip failure at the short anchorage end. This indicated that the experimental data were stable, and the results were reliable.

**Figure 8:**
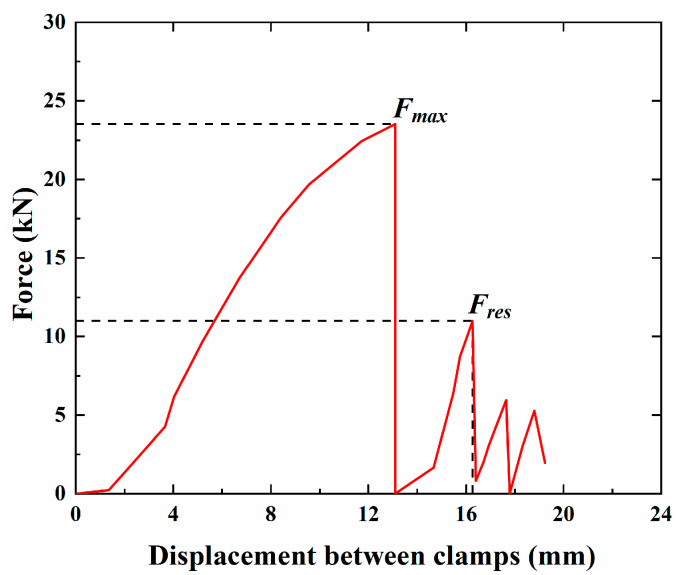
Force–displacement curve of CFRP tendon for benchmark specimen BS-t1-A.

Figure 8 shows the pull-out force–displacement curve of a representative specimen (BS-t1-A). Before reaching the ultimate state, there was minimal relative slip between the CFRP anchorage and LTM. Upon reaching the ultimate state, a distinct cracking sound could be heard. Due to the universal testing machine’s inability to handle the sudden change in tensile force, the tensile force abruptly dropped to zero, causing the machine to automatically stop. After restarting the machine, the specimen continued to bear the load, but its maximum load capacity decreased compared to the initial stage. This peak force is referred to as the residual bonding force $“F_{res}”$. Subsequently, the pull-out force–displacement curve exhibited a stepped shape, with the pull-out force decreasing as the slip increased.

By substituting the radial compressive stress that is applied in the reference test, the theoretical residual bond strength is calculated to be 15.796 MPa, with an 11.8% deviation from the experimental results, which is within 15%, and the residual bond strength meets expectations.

### 4.2. Experimental Group

**Figure 9:**
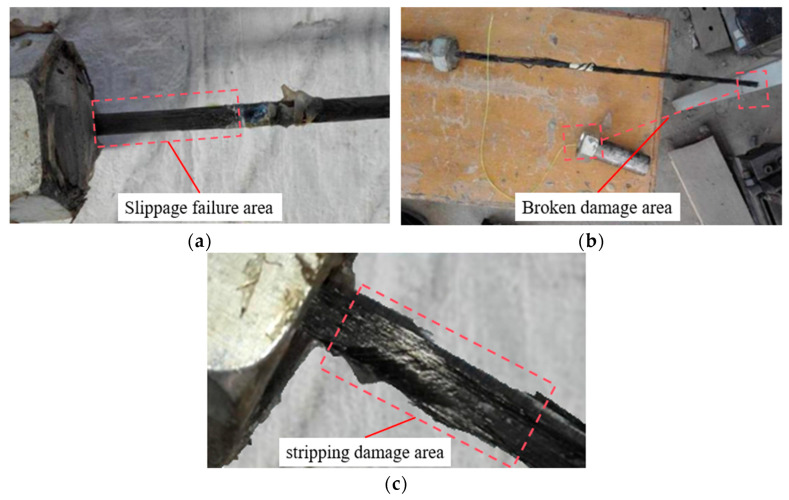
Shape of specimen damage and final colloidal morphology after temperature action: (**a**) T50-t5-A short-anchorage end slippage; (**b**) T60-t7-A long-anchorage end breakage; (**c**) T70-t5-C short-anchorage end stripping damage.

**Figure 10:**
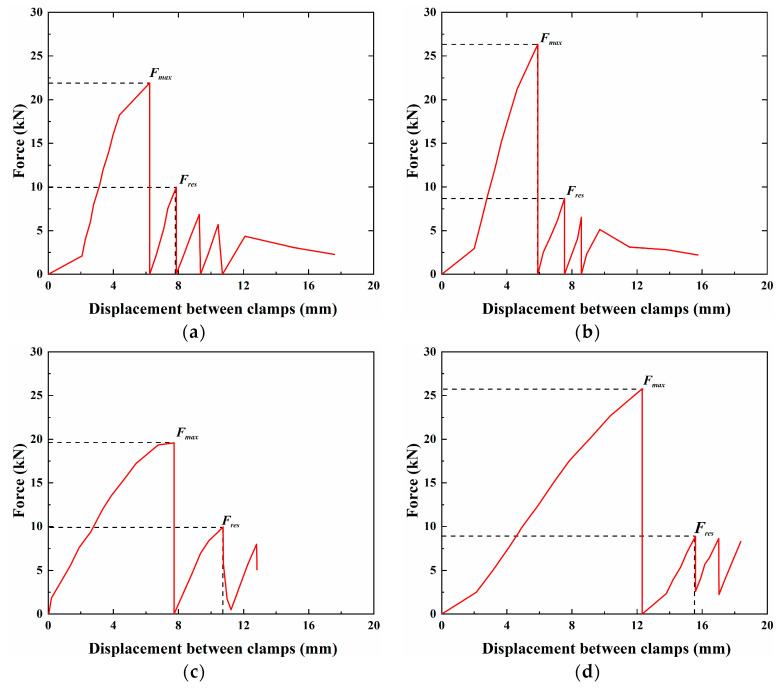
Extraction force–displacement curves of CFRP reinforcement in specimen after temperature action: (**a**) Specimen T60-t1-C; (**b**)Specimen T60-t7-C; (**c**) Specimen T70-t1-C; (**d**) Specimen T70-t5-A.

Table 5 summarizes the ultimate bearing capacity $F_{max}$, maximum bond strength $\tau_{max}$, maximum bond strength degradation rate $k_{1}$, residual bearing capacity $F_{res}$, residual bond strength $\tau_{res}$, and residual bond strength degradation rate $k_{2}$, as well as the failure modes of each specimen. Figure 9 illustrates the failure modes of the specimens, which include sliding failure, delamination failure, and fracture failure. Sliding failure occurred when the bond strength at the CFRP–reinforcement interface reached its limit, delamination failure was caused by variability in the material quality of the CFRP itself, and fracture failure resulted from non-axial forces acting on the CFRP during loading. The specimens T60-t1-B, T60-t5-A, T60-t7-A, T70-t5-B, and T70-t7-B experienced fracture failure and were excluded from the calculations of the maximum bond strength. Subsequent observations of the gel in specimens T50-t7-C and T70-t1-B revealed numerous bubbles at the short anchorage end, leading to significant differences in their bond strength compared to equivalent parallel specimens. In contrast, the gel density in other specimens was more consistent, and the force–displacement curves of some representative specimens are shown in Figure 10.

**Figure 11:**
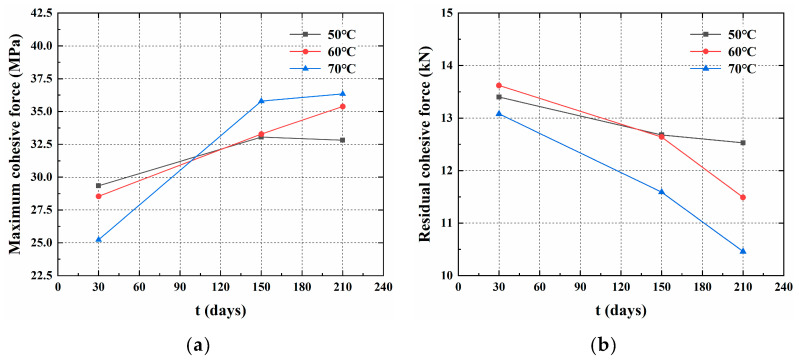
Time–bond strength curve under different temperature conditions: (**a**) maximum bond strength; (**b**) residual bond strength.

As shown in Figure 11 and Table 5, after 30 days of aging, the maximum bond strength $\tau_{max}$ showed a decreasing trend with the increasing aging temperature. The decrease was gradual in the 50 °C to 60 °C range and became more pronounced between 60 °C and 70 °C. In contrast, after 150 and 210 days of aging, the maximum bond strength $\tau_{max}$ increased with the rising temperature. This phenomenon was primarily related to the epoxy resin that was used as the bonding medium in the specimens. The epoxy resin was temperature-sensitive; thus, after 30 days of aging, its adhesive strength decreased with the increasing temperature. As the aging time increased, the polymer within the gel underwent an aging reaction, which increased its brittleness at room temperature, resulting in a higher shear strength. After 150 and 210 days of aging, the maximum bond strength stabilized.

Compared to the reference specimens, the residual bond strength continued to decrease over time under different temperature conditions. Table 5 and Figure 11 indicate that after 30 days of aging at 50 °C, 60 °C, and 70 °C, the degradation rates of the residual bonding strength were 3.72%, 2.16%, and 6.02%, respectively. After 150 and 210 days, varying degrees of degradation were observed, which were particularly significant at 70 °C. Longer aging times correlated with a lower residual bonding strength, although the rate of decline diminished. The residual bonding strength $\tau_{res}$ was primarily composed of adhesive and frictional forces. After gel aging, it exhibited a higher shear strength and became more brittle, resulting in a decrease in adhesive and frictional forces, which led to a reduced bonding strength at the CFRP-LTM interface. This phenomenon explained the decrease in residual bond strength at the same time as the increase in maximum bond strength.

## 5. Long-Term Durability Prediction

### 5.1. Durability Prediction Theory

Durability generally refers to the ability of a CFRP anchorage system to maintain its mechanical properties and bond strength over long-term use. Durability is directly related to strength degradation after thermal aging, as the temperature accelerates the degradation process of the material, thereby reducing its reliability and stability during prolonged use. After understanding the interface mechanisms of CFRP–epoxy resin, long-term predictions of the residual bond strength of CFRP-bonded anchorage systems under high-temperature conditions can be made. The Arrhenius theory is commonly used to study the degradation trends of composite materials in adverse environments. Its extrapolation method can establish a predictive model for the aging life of composites to examine their performance under hygrothermal aging [34]. Thus, the Arrhenius theory is applied to predict the retention rate of the residual bond strength of anchorages under different temperature conditions. The Arrhenius expression is given by the following equation [35]:

(5) $$k=A\exp\left(-E_{a}∕RT\right),$$

where k represents the decay rate (1/time), *A* is a constant, $E_{a}$ is the activation energy, R is the universal gas constant, and T is the temperature in Kelvin.

Equation (5) can also be expressed as follows:

(6) $$\frac{1}{k}=\frac{1}{A}\exp\left(E_{a}∕RT\right),$$

Or

(7) $$\ln\left(\frac{1}{k}\right)=\frac{E_{a}}{R}\cdot \frac{1}{T}-lnA.$$

In Equation (6), the variable *k* is defined as the reciprocal of the time that is required for the material properties to reach a specific level. Equation (7) demonstrates that the logarithm of the time that is required for the material properties to reach a specific value is linearly related to 1/*T*, showing the slope of $E_{a}$/*R*.

From Equation (7), the time shift factor (TSF) that is required for the tensile strength to reach the same value (denoted as c) at temperatures $T_{0}$ and $T_{1}$ can be expressed as follows:

(8) $$\mathrm{TSF}=\frac{t_{0}}{t_{1}}=\frac{c/k_{0}}{c/k_{1}}=\frac{k_{1}}{k_{0}}=\frac{A\exp(-E_{a}/RT_{1})}{A\exp(-E_{a}/RT_{0})}=\exp\left[\frac{E_{a}}{R}\left(\frac{1}{T_{0}}-\frac{1}{T_{1}}\right)\right],$$

where one day at temperature $T_{1}$ is equivalent to TSF days at temperature $T_{0}$.

### 5.2. Durability Prediction Results

Before applying the Arrhenius theory for long-term predictions, it is essential to consider data degradation [36]. A commonly used degradation model is the exponential model, which can be expressed as follows:

(9) $$R=100\exp\left(-T/a\right),$$

where *R* is the retention rate of anchorage capacity, *T* is the time of exposure, and a is the fitting parameter.

**Figure 12:**
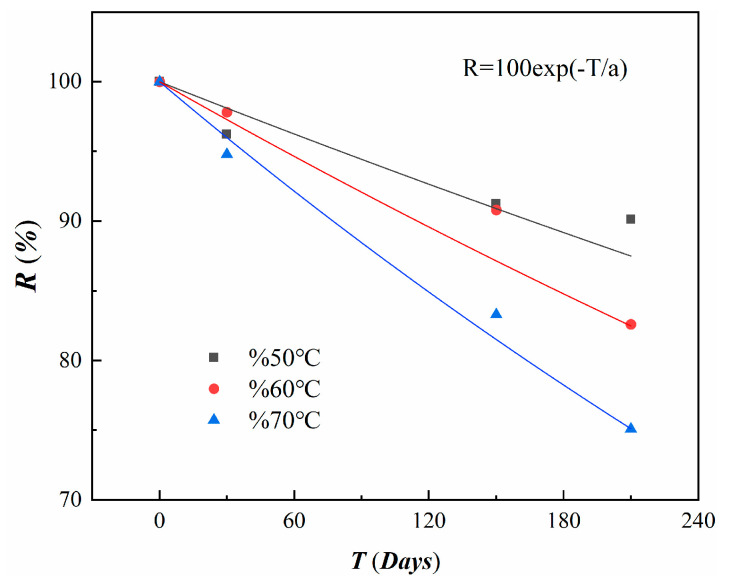
Degradation fitting curve of anchorage performance with time.

**Figure 13:**
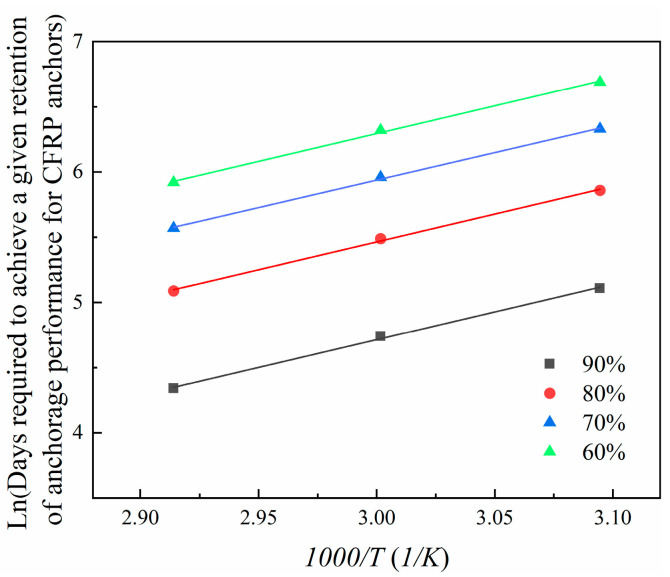
Arrhenius plots of anchorage performance of CFRP anchorages.

This experiment employs Equation (9) for degradation analysis, with the results shown in Figure 12 and corresponding parameters listed in Table 6. Under the conditions of 50 °C, 60 °C, and 70 °C, the degradation times that are required for the anchorage performance to reach retention rates of 60%, 70%, 80%, and 90% can be derived using Equation (9). Subsequently, data analysis is performed using Equation (7). As shown in Figure 13, the fitted lines are nearly parallel, consistent with the Arrhenius relationship. The slope $\left(E_{a}/R\right)$, correlation coefficient $\left(R^{2}\right)$, and constant lnA are provided in Table 7.

By using Equation (8) and the Arrhenius plot, the time shift factor (TSF) that is required to achieve an equivalent anchorage capacity retention rate (denoted as c) at temperatures $T_{1}$ and $T_{0}$ can be determined. Relevant meteorological data from the National Climatic Data Center (NCDC) [37] were obtained, and the annual average temperatures of several representative cities were listed. The TSF corresponding to the annual average temperatures of these cities was calculated and is presented in Table 8.

**Figure 14:**
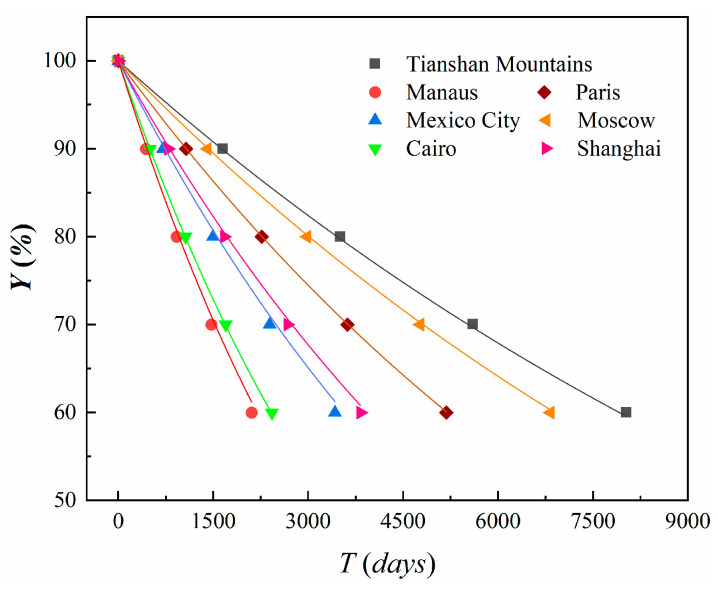
Long-term degradation rate prediction of CFRP anchorage performance in representative regions.

The retention rate curve for the CFRP anchorages is shown in Figure 14, with the corresponding coefficients a and $R^{2}$ being presented in Table 9. According to the prediction results, the rate of decline in anchorage capacity for CFRP anchorages is fastest in the rainforest region represented by Manaus and slowest in the plateau mountainous region represented by the Tianshan Mountains. Table 10 summarizes the degradation data for CFRP cable anchorage devices after 10, 30, and 50 years in regions with average annual temperatures of 0 °C, 10 °C, and 20 °C. From Table 10, it can be seen that after 10 years, the degradation rates were 18.35%, 30.14%, and 44.5% for the 0 °C, 10 °C, and 20 °C regions, respectively. After 50 years, the degradation rates increased to 63.72%, 83.36%, and 94.73%. Therefore, the temperature significantly affects the long-term anchorage performance of CFRP cable bonding devices. In regions with higher temperatures, the design of the anchorage devices needs to be more conservative.

## 6. Conclusions

This study investigated the degradation of bonding performance at the CFRP cable-bonded anchorage interface under prolonged temperature exposure. The key findings are as follows:

1. After 30 days at 50 °C, 60 °C, and 70 °C, the maximum bonding strength degraded by 6.02%, 8.58%, and 19.18%, respectively. However, after 150 and 210 days, the bonding strength increased by 5.86%, 6.60%, and 14.63% and 5.09%, 13.29%, and 16.39%, respectively.

2. The residual bonding strength continuously decreased, with degradation rates of 3.72%, 2.16%, and 6.02% after 30 days at 50 °C, 60 °C, and 70 °C. After 210 days, the rates increased to 9.97%, 17.45%, and 24.88%.

3. Higher temperatures and longer exposure times lead to faster degradation, but the rate of degradation slows over time, eventually stabilizing.

4. In regions with average annual temperatures of 0 °C, 10 °C, and 20 °C, after 10, 30, and 50 years, the anchorage capacity degrades by 18.35%, 45.57%, and 63.72%; 30.14%, 65.90%, and 83.36%; and 44.50%, 82.90%, and 94.73%, respectively.

These findings highlight the significant long-term impact of temperature on CFRP anchorage systems, necessitating more conservative designs in regions with higher temperatures.

Based on the findings of this study, the following directions for further research are proposed to enhance the long-term durability of CFRP cable anchorages under different temperature conditions and improve their application in engineering.

1. Temperature–Humidity Interaction Study: Future research should investigate the combined effects of temperature and humidity on the mechanical performance of CFRP cable anchorages. Understanding how water absorption and temperature fluctuations interact will provide valuable insights into the performance of CFRP anchorages in real-world environments.

2. Combined Effects of Thermal Aging and Dynamic Loading: In practice, CFRP cable anchorages face both high temperatures and dynamic loads. Future studies should examine how thermal aging combined with dynamic loading affects the long-term performance of these anchorages, using both experimental testing and numerical simulations.

3. Design Optimization for Enhanced Durability: Future research should focus on optimizing the design of CFRP cable anchorages to improve their durability under high-temperature conditions. This includes exploring modifications to their geometry, interface treatments, and use of advanced materials to enhance the thermal stability and resistance to aging.

By addressing these areas, future studies can contribute to developing more reliable and durable CFRP cable anchorage systems for long-term use in high-temperature environments.

## Figures and Tables

**Table 1:** Material properties of CFRP anchorages.

Design Diameter (mm)	Measured Diameter (mm)	Superficial Form	Tensile Strength (MPa)	Elastic Modulus (GPa)
5.0	4.8	Embossing	2322	135

Note: The material data were provided by the manufacturer.

**Table 2:** Material properties of epoxy resin adhesive.

Shear Strength (MPa)	Tensile Strength (MPa)	Compressive Strength (MPa)	Shelf Life (min)	Viscosity (mPa·s)	Elongation at Break (%)
18.33	41.17	54.88	78	330	11.3

Note: The material data were provided by the manufacturer.

**Table 3:** Setting of each working condition and specimen number.

Working Condition			Specimen Identification		
Baseline Specimen			BS-t1-A	BS-t1-B	BS-t1-C
-	Temperature (°C)	Aging Duration (Day)	-	-	-
Temperature	50 °C	30	T50-t1-A	T50-t1-B	T50-t1-C
		150	T50-t5-A	T50-t5-B	T50-t5-C
		210	T50-t7-A	T50-t7-B	T50-t7-C
	60 °C	30	T60-t1-A	T60-t1-B	T60-t1-C
		150	T60-t5-A	T60-t5-B	T60-t5-C
		210	T60-t7-A	T60-t7-B	T60-t7-C
	70 °C	30	T70-t1-A	T70-t1-B	T70-t1-C
		150	T70-t5-A	T70-t5-B	T70-t5-C
		210	T70-t7-A	T70-t7-B	T70-t7-C

Note: In this study, each specimen was identified by a specific acronym. The first element denotes the environmental conditions: “BS” stands for baseline specimen, while “T50”, “T60”, and “T70” represent temperatures of 50 °C, 60 °C, and 70 °C, respectively. The second element indicates the aging time: “t1”, “t5”, and “t7” represent 30 days, 150 days, and 210 days, respectively. The third element distinguishes between specimens under the same conditions, with “A”, “B”, and “C” indicating three distinct specimens.

**Table 4:** Pull-out test results for benchmark specimens.

No.	$F_{\max}$ (kN)	$\tau_{\max}$ (MPa)	$F_{\mathrm{res}}$ (kN)	$\tau_{\mathrm{res}}$ (MPa)	Damage Pattern
BS-t1-A	23.5	31.23	10.99	13.92	Short-anchorage end slippage
BS-t1-B	23.3		10.63		Short-anchorage end slippage
BS-t1-C	23.6		9.87		Short-anchorage end slippage

**Table 5:** Results of pull-out test after temperature-accelerated aging.

No.	$F_{max}$ (kN)	$\tau_{\max}$ (MPa)	$k_{1}$ (%)	$F_{res}$ (kN)	$\tau_{res}$ (MPa)	$k_{2}$ (%)	Damage Pattern
T50-t1-A	24.23	29.35	−6.02	10.35	13.40	−3.72	Short-anchorage end slippage
T50-t1-B	20.88			9.65			Short-anchorage end slippage
T50-t1-C	21.24			10.32			Short-anchorage end slippage
T50-t5-A	25.58	33.06	5.86	9.78	12.68	−8.96	Short-anchorage end slippage
T50-t5-B	22.32			9.98			Short-anchorage end slippage
T50-t5-C	26.84			8.91			Short-anchorage end slippage
T50-t7-A	24.28	32.82	5.09	9.24	12.53	−9.97	Short-anchorage end slippage
T50-t7-B	25.18			9.66			Short-anchorage end slippage
T50-t7-C	20.52			-			Long-anchorage end slippage
T60-t1-A	21.25	28.55	−8.58	10.57	13.62	−2.16	Short-anchorage end slippage
T60-t1-B	26.22			-			Broken end of short anchorage
T60-t1-C	21.78			9.97			Short-anchorage end slippage
T60-t5-A	29.44	33.29	6.60	-	12.64	−9.61	Broken end of long anchorage
T60-t5-B	24.36			9.68			Short-anchorage end slippage
T60-t5-C	25.82			9.38			Short-anchorage end slippage
T60-t7-A	29.16	35.39	13.29	-	11.49	−17.45	Broken end of long anchorage
T60-t7-B	27.18			8.65			Short-anchorage end slippage
T60-t7-C	26.16			8.68			Short-anchorage end slippage
T70-t1-A	18.14	25.24	−19.18	9.78	13.08	−6.02	Short-anchorage end slippage
T70-t1-B	14.86			-			Long-anchorage end slippage
T70-t1-C	19.90			9.95			Short-anchorage end slippage
T70-t5-A	25.76	35.80	14.63	8.92	11.59	−16.73	Short-anchorage end slippage
T70-t5-B	30.32			-			Broken end of long anchorage
T70-t5-C	28.20			8.56			Short-anchorage end stripping
T70-t7-A	27.30	36.35	16.39	7.79	10.46	−24.88	Short-anchorage end slippage
T70-t7-B	29.76			-			Broken end of long anchorage
T70-t7-C	27.48			7.98			Short-anchorage end slippage

Note: In Table 5, $\tau_{\max},\;\tau_{res},\;k_{1},$ and $k_{2}$ are taken as the averages of the valid experimental trials. A negative value of k indicates a decrease in intensity and vice versa for an increase in intensity.

**Table 6:** Fitting parameters for residual bond.

Temperature (°C)	$R=100\exp\left(-T/a\right)$	
	a	$R^{2}$
50	1573	0.83
60	1091	0.93
70	734	0.98

**Table 7:** Correlation coefficients in the Arrhenius plot.

Retention (%)	$E_{a}/R$	lnA	$R^{2}$
90	4265	8.081	0.998
80	4265	7.331	0.998
70	4265	6.692	0.999
60	4265	6.500	0.998

**Table 8:** TSFs under different environmental conditions.

Location	Annual Mean Temperature (°C)	TSF		
		50 °C	60 °C	70 °C
Tianshan Mountains	2.0	9.99	14.86	21.57
Manaus	28.6	2.62	3.89	5.66
Mexico City	18.0	4.26	6.34	9.21
Cairo	25.0	3.02	4.49	6.52
Paris	10.0	6.45	9.59	13.93
Moscow	4.9	8.50	12.64	18.35
Shanghai	15.8	4.76	7.08	10.29

**Table 9:** Coefficients of the fitting curves.

Location	a	$R^{2}$
Tianshan Mountains (2.0)	15,500	0.99
Manaus (28.6)	4286	0.99
Mexico City (18.0)	6984	0.99
Cairo (25.0)	4732	0.99
Paris (10.0)	10,177	0.99
Moscow (4.9)	13,500	0.99
Shanghai (15.8)	7689	0.99

Note: The numbers in parentheses in the table represent the local annual average temperature in degrees Celsius (°C).

**Table 10:** Long-term prediction of degradation in anchorage load capacity of CFRP cable anchorages in different temperature regions.

Temperature (°C)	Degradation Rate After 10 Years (%)	Degradation Rate After 30 Years (%)	Degradation Rate After 50 Years (%)
0	18.35	45.57	63.72
10	30.14	65.90	83.36
20	44.50	82.90	94.73

## Data Availability

The original contributions presented in this study are included in the article. Further inquiries can be directed to the corresponding author.
